# Theranostic quantum dots for crossing blood–brain barrier *in vitro* and providing therapy of HIV-associated encephalopathy

**DOI:** 10.3389/fphar.2013.00140

**Published:** 2013-11-15

**Authors:** Gaixia Xu, Supriya Mahajan, Indrajit Roy, Ken-Tye Yong

**Affiliations:** ^1^College of Optoelectronic Engineering, Key Laboratory of Optoelectronics Devices and Systems of Ministry of Education/Guangdong Province, Shenzhen UniversityShenzhen, China; ^2^Division of Allergy, Immunology and Rheumatology, Department of Medicine, The State University of New York at BuffaloBuffalo, NY, USA; ^3^Department of Chemistry, University of DelhiNew Delhi, India; ^4^School of Electrical and Electronic Engineering, Nanyang Technological UniversitySingapore, Singapore

**Keywords:** theranostic quantum dots, HIV-associated encephalopathy, *in vitro* blood–brain barrier model, nanomedicine, drug delivery

## Abstract

The blood–brain barrier (BBB) is a complex physiological checkpoint that restricts the free diffusion of circulating molecules from the blood into the central nervous system. Delivering of drugs and other active agents across the BBB is one of the major technical challenges faced by scientists and medical practitioners. Therefore, development of novel methodologies to address this challenge holds the key for both the diagnosis and treatment of brain diseases, such as HIV-associated encephalopathy. Bioconjugated quantum dots (QDs) are excellent fluorescent probes and nano-vectors, being designed to transverse across the BBB and visualize drug delivery inside the brain. This paper discusses the use of functionalized QDs for crossing the blood–brain barrier and treating brain disease. We highlight the guidelines for using *in vitro* BBB models for brain disease studies. The theranostic QDs offers a strategy to significantly improve the effective dosages of drugs to transverse across the BBB and orientate to the targets inside the brain.

## INTRODUCTION

**FIGURE 1:**
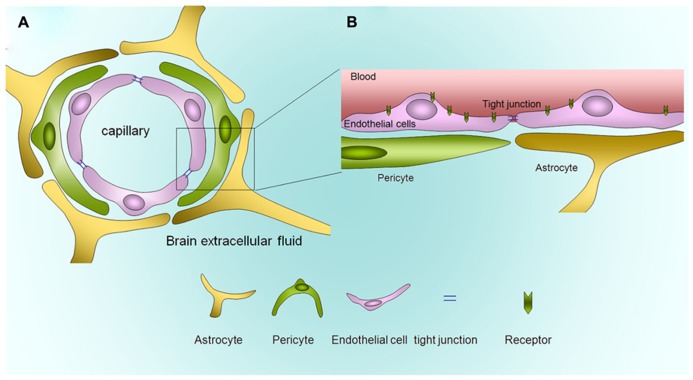
**Anatomical structure of the blood–brain barrier. (A)** The diagram of blood–brain barrier *in vivo*. **(B)** Magnification of the BBB. The brain endothelium forms the BBB.

The blood–brain barrier (BBB) dynamically responds to physiological and pathophysiological events that lead to pathogenesis and progression of many neurological disorders (Banks, 1999; Banks et al., 2006). BBB works as a critical checkpoint between the central nervous system (CNS) and the peripheral circulation, as **Figure 1**, which prevents harmful substances from entering the brain, but allows the essential nutrients to enter (Schinkel, 1999). The transporters that help the uptake by the BBB include GLUT-1, a glucose carrier, and L1, an amino acid transporter, while the BBB reject foreign substances by an efflux mechanisms that uses efflux transporters such as *P*-glycoprotein (Fromm, 2000; Sun et al., 2003). However, this barrier also blocks therapeutic drugs to reach the pathological tissues behind BBB (Abbott, 2002; Abbott et al., 2010; Abbott and Friedman, 2012). Since the BBB limits the brain penetration of most CNS drug candidates, neurological disorders such as HIV-associated encephalopathy (HIVE), has significant morbidity and mortality (Berger and Avison, 2004; Alexaki et al., 2008; Strazza et al., 2011).

The BBB is a dynamic interface that controls the influx and efflux of a wide variety of substances, including endogenous nutrients and exogenous compounds such as drugs, thereby maintaining a homeostatic environment for the CNS (Ballabh et al., 2004; Weiss et al., 2009). Under normal physiological conditions, BBB prevents transport of bacteria, large molecules, and most small molecules into the brain. To be BBB permeable, molecules need to be lipid soluble, and less than 400 Da in size. Larger biomolecules, which are unable to cross the BBB due to their size and polarity, could pass through the BBB if they are transported by receptor-mediated transcytosis (RMT) using ligands that bind to specific BBB receptors. There are more than 7000 drugs in the comprehensive medicinal chemistry database, and only 5% of these drugs can be used to treat the CNS diseases, with the most predominant being for the treatment of depression, schizophrenia, and insomnia (Ghose et al., 1999). In a related study, 12% of all drugs are shown to be active in the CNS, but only 1% of all drugs are active in the brain for diseases other than affective disorders (Lipinski, 2000). The importance of developing new approaches to brain drug development is illustrated by considering the limitations of the existing brain drug delivery strategies. These delivery systems include transcranial brain drug delivery, trans-nasal brain drug delivery, BBB disruption, and small molecule lipidization (Pardridge, 2012; Pardridge and Boado, 2012). Alternatively, lipid carriers are attached to water-soluble drugs. However, in actual practice, the reformulation of a water soluble drug with lipidization modifications is difficult to execute successfully. Currently, there is not a single example of a drug presently sold whereby medicinal chemistry was successfully used to convert non-brain-penetrating drug into a molecule that crosses the BBB in pharmacologically significant amounts.

Fortunately, nanotechnology offer great promise for overcoming the limited penetration of drugs through the BBB (Jain, 2012). Over the last two decades, nanoparticle research has focused on the development of new drug delivery vehicles (Bruchez et al., 1998; Ghose et al., 1999; Prasad, 2004). New nanomaterials, including QDs, gold nanoparticles, carbon nanotubes, ultra-sensitive nanosensors, smart nanomaterials, molecular motors, shape memory nanomaterials, and biological templates for nanoscale devices, had been investigated in nanomedicine (Kubitschko et al., 1997; Titus and Gilbert, 1999). It is known that the numerous drugs, though being highly efficacious, are limited in their therapeutic applications owing to their poor solubility and stability in physiological medium (Davis, 1997). Nanoparticles not only enable the development of stable aqueous formulation of such drugs, but also help in their bioavailability and site-specific delivery, using biotargeting approaches (Uhrich et al., 1999). In addition, nanoparticles also can be designed for the sustained or externally controlled release of these drugs from the particle matrix within the target site of interest (Kreuter, 2004). Furthermore, these enhanced drug-delivery nanoplatforms can be combined with diagnostic probes, as well as designed to target “hard-to-reach” diseased sites. For example, the transferrin (Tf) is a type of Fe-binding glycoprotein and they could be easily used to conjugate with nanoparticles for targeting the BBB and facilitate the RMT process. Tf receptor (TfR) mediates cellular uptake of iron bound to Tf. Upon binding of Tf to its receptor, the receptor-ligand complex is endocytosed via clathrin-coated vesicles and occurs in 3 steps (1) receptor-mediated endocytosis of the compound at the luminal (blood) side, (2) movement through the endothelial cytoplasm, and (3) exocytosis at the abluminal (brain) side of the brain capillary endothelium. These multimodal nanoparticles would be ideal candidates for curing such diseases which are currently untreatable using standard chemo/radio-therapeutic and surgical approaches.

Though a number of approaches have been used for delivery of liposomes containing drugs and genes across the BBB in small animals, only handful of reports of nanoparticle delivery using immunotargeting approach can be found in the literature (Pardridge, 2012; Pardridge and Boado, 2012). Thus, there is a great interest in the nanomedicine field to develop optimized traceable drug-loaded nanoparticle formulations that can be safely used to facilitate drugs across the BBB for treating the brain diseases, and at the same time visualize the distribution profile of the nanoparticles in the brain. Therefore, in this review, the development of functionalized fluorescent QDs for crossing the blood–brain barrier and treating brain disease will be discussed and presented. The guidelines for using *in vitro* BBB models for brain disease studies will be highlighted. This review is intended to discuss the current developments of QD bioconjugates for transmigration across BBB and therapy of brain diseases.

## QUANTUM DOTS IN NANOMEDICINE APPLICATIONS

Quantum dots are semiconductor nanocrystals that have sizes ranging from 2 to 10 nm. In general, it is well documented that the emission wavelength of the QDs can be tuned from 450 to 1800 nm by manipulating their size, shape, and composition of the nanocrystals. QDs possess a few unique optical properties. For example, they have excellent resistance to photo-bleaching, large absorption cross section, relatively long fluorescence lifetime, and good quantum yield that can be as large as 70–80% (Yong et al., 2009b). The narrow emission and broad excitation spectra make it possible to trace the dynamics of several interested molecules *in vivo* or *in vitro* (Gao et al., 2005). In addition, the large two photon absorption cross section of QDs, compared to organic dyes, makes them very promising optical agents for two-photon laser excitation, which can be performed in the near-infrared window with better imaging penetration depth in tissue (Hilderbrand and Weissleder, 2010; Yong et al., 2011). These properties make QDs very attractive for biophotonics and nanomedicine research applications.

**FIGURE 2:**
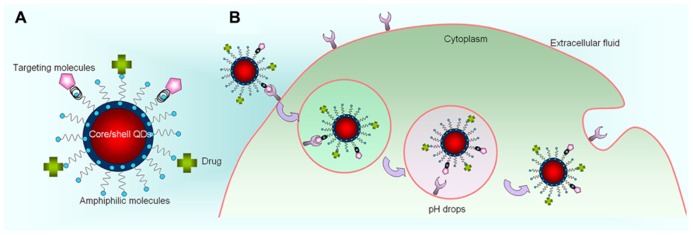
**Functionalized QDs and its theranostic mechanism at cellular level. (A)** Theranostic QDs. **(B)** The dynamics of QDs-based nanoplex inside living cells.

To date, colloidal core/shell QDs such as CdSe/ZnS, CdSe/ZnCdS, CdTe/CdSe, and InP/ZnS are being commonly used in biological and medical research (Michalet et al., 2005; Pinaud et al., 2010). Among many methods available in the literature for synthesizing QDs, hot colloidal synthesis approach remains the most promising approach for obtaining robust QDs for nanomedicine applications. For core QDs such as CdSe and CdTe, they can be prepared at high temperature by the reaction between cadmium oxide dissolved in oleic acid and trioctylphosphine (TOP) and/or TOP-Selenium. The reaction resulted in the formation of mono-dispersed QDs. However, synthesized QDs are unable to be employed in biological applications unless the following critical challenges are addressed (**Figure 2**).

First, it is important to passivate the core QD with a thin layer of high band gap materials, such as ZnS. There are many advantages for core/shell QDs upon comparing with unpassivated ones. For example, it was discovered that the chemical and optical stabilities of QDs can be maintained when the QDs are passivated with higher band-gap semiconductor materials. Also, the shells will significantly reduce the QDs toxicity, which makes them amenable for biological applications (Hedi et al., 2000). Although many types of core/shell QDs were fabricated, only CdSe/ZnS, CdTe/ZnS, and CdSe/CdS/ZnS were found to be useful for *in vivo* imaging applications. However, there does exist toxicity concerns about CdSe and CdTe QDs due to the presence of cadmium, and further studies are needed to resolve these challenges. In recent years, many research teams have prepared cadmium-free QDs such as InP, CuInS_2_, AgInS_2_, and silicon for biological applications (Erogbogbo et al., 2008; Yong et al., 2009a, 2010; Liu et al., 2013).

Second, general high quality core/shell QDs are prepared by using hot colloidal synthesis and its surface is passivated with hydrophobic moieties, which prohibit them to be dispersible in aqueous phase. Thus, water-dispersible QDs with reactive functional groups are needed for *in vitro* and *in vivo* applications. The techniques for transferring organically dispersible core/shell QDs from organic phase to aqueous phase have been extensively studied for the last decade. In general, the approaches used for fabricating water-dispersible QDs includes (i) functionalizing QD surface with amphiphilic molecules such as mercapto acids, and hydrophilic dendrimers; and (ii) coating the QDs with biocompatible surface layer such silica-shell and amphiphilic polymers. For example, Chan et al. demonstrated the preparation of water dispersible CdSe/ZnS QDs for *in vitro* imaging by functionalizing the QD surface with mercaptoacetic acid through ligand exchange method (Bruchez et al., 1998; Chan and Nie, 1998). Using a similar approach, Uyeda et al. demonstrated that water-dispersible QDs can be prepared by functionalizing the QD surface with the bidentate dihydrolipoic acid (DHLA) (Uyeda et al., 2005). To date, surface modification of QDs using mercapto ligands has remained a popular method for preparing water dispersible QDs (Medintz et al., 2005).

Third, conjugation of biomolecules to the surface of water-dispersible QDs is another important requirement for *in vitro* and *in vivo* targeted delivery applications. Biomolecules such as antibodies, nucleic acids, peptides, and aptamers can be attached to the QD surface by either covalent or non-covalent interactions. Commonly, conjugation of proteins, antibodies, peptides, and drug molecules to the QD surface is required for targeted delivery to the area of interest. The QD surface can be decorated with functional groups such as carboxylic acid, primary amine, and thiol, which can be used for conjugation with targeting ligands by using conjugation chemistries such as carbodiimide, maleimide, and succinimide. Avidin-biotin cross-linking technique is another popular method for conjugating biomolecules on the surface of QDs.

## QUANTUM DOTS IN HIV-ASSOCIATED ENCEPHALOPATHY THERAPY

### *IN VITRO* MODELS OF THE HUMAN BBB

The BBB is a critical interface and acts as a physical and metabolic barrier between the CNS and the peripheral circulation that serves to regulate and protect the microenvironment of the brain. The primary function of the normal BBB is to establish and maintain homeostasis in the CNS (Bradbury, 1993; Abbott and Friedman, 2012). The BBB is not rigid and comprises of dynamic vessels that are capable of responding to rapid changes in the brain or blood. It is composed of specialized brain capillary endothelial cells (e.g., BMVECs) and astrocytic end-feet that enhance the differentiation of BBB endothelium (Abbott, 2002; Abbott et al., 2010; Abbott and Friedman, 2012). The BBB has at least three types of cell-to-cell junction structures between adjacent endothelial cells and/or astrocytes, which are the gap junctions, adherens junction, and the tight junctions. The high expression of tight junction proteins is a special characteristic of the BBB (de Lange, 2012; Luissint et al., 2012).

**FIGURE 3:**
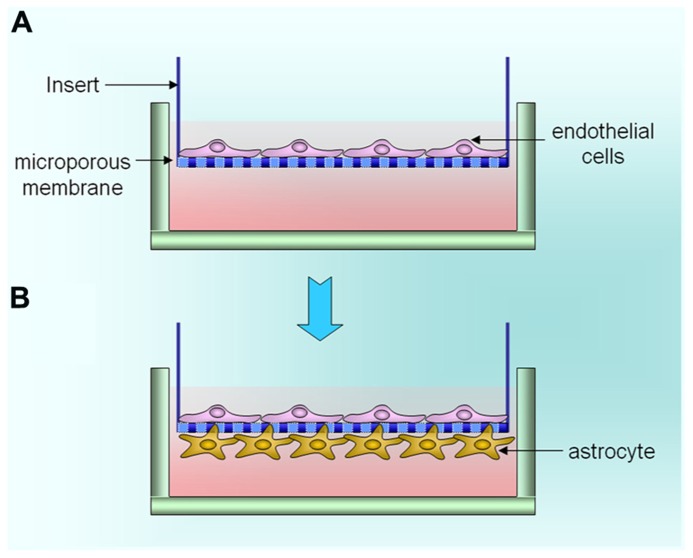
**The development of *in vitro* BBB model. (A)** Monolayer *in vitro* BBB. **(B)** Bilayer *in vitro* BBB.

Animal experiments for drug screening of brain disease are time-consuming and costly, which seriously impedes the brain drug development. There is a considerable interest in establishing *in vitro* BBB cell culture models for a few reasons such as studying drugs that penetrate the BBB and understanding how abnormality of the BBB is correlated with the pathogenesis of various neurological diseases (Stanness et al., 1996). A good *in vitro* BBB model must reproduce the salient features of the *in vivo* BBB and also allow one to manipulate the system to mimic the neuropathogenic process. Fortunately, several *in vitro* tissue culture systems have been developed to reproduce the physical and biochemical properties of the intact BBB (Stanness et al., 1999; Cucullo et al., 2002, 2005; Santaguida et al., 2006; Naik and Cucullo, 2012). Many groups have employed a setup using a porous membrane support as substrate for culturing BMVEC *in vitro* that is submerged in culture media (**Figure 3A**). This system is characterized by side-to-side diffusion, is capable of inducing polarity in the endothelial cells and permits study of bidirectional transendothelial transport of solutes (Joo, 1993).

A major disadvantage of this system is the lack of physiologic shear stress that induces tight junction qualities in BMVEC. Therefore, it is generally reported that the tightness of the barrier in this type of model is much less stringent than that of the *in vivo* BBB. A dynamic, three dimensional *in vitro* culture system (DIV-BBB) is more relevant to the *in vivo* BBB phenotype than other models (Cucullo et al., 2011; Naik and Cucullo, 2012). In this model, the endothelial cells are cultured with astrocytes across a membrane with pore size of 0.1–0.5 μm, under pulsate flow (**Figure 3B**). In general, this model is not suitable for examining the transmigration process because of the extremely small size of the membrane pores. The advantages of this model are the presence of sheer stress, easier regulation of nutrients and gases, and closest model mimicking the *in vivo* BBB. The co-culture model has been extensively used to date because it can be conveniently and reproducibly formed in large quantities. More importantly, the system provides detailed information about the cellular and molecular mechanisms of a wide variety of neurological disease conditions. The use of astrocyte co-culture and conditioned media have been reported to reduce the permeability of the BMVEC (Rubin et al., 1991). Astrocytes also may influence the expression of adhesion molecules by the BMVEC that form the BBB. The Persidsky model uses a 3.0-μm diameter polyethylene terephthalate (PET) membrane insert, while other investigators use a 0.45-μm diameter PET membrane insert (Persidsky et al., 1997; Mukhtar and Pomerantz, 2000). In our study, we have validated the transwell co-culture model that provides an opportunity to examine the expression of tight junction protein, permeability, and transmigration of different leukocyte populations under different experimental stimuli. Thus, we propose that, although some model systems may be better than others, the most important aspect of any study is the accuracy and reproducibility of the model and its ability to allow manipulations in mimicking disease processes.

### GENE SILENCING USING QD/SIRNA NANOPLEX DELIVERY TO MAINTAIN THE INTEGRITY OF THE BLOOD–BRAIN BARRIER

In neuroinflammatory conditions, such as multiple sclerosis, HIVE, meningitis, cerebral ischemia, and brain tumors, the matrix-degrading metalloproteinases (MMP) play an important role in disrupting the BBB. MMPs are proteolytic enzymes that are instrumental in the turnover of the extracellular matrix (ECM), and are mediators of cell migration. MMP-9 is known to mediate the transmigration of inflammatory leukocytes across basement membranes, thus leading to proteolytic degradation of ECM components. In addition to degrading the neurovascular matrix, MMP-9 promotes neuronal apoptosis by disrupting cell-matrix interactions that enhance the permeability of the BBB, thereby exacerbating neurological diseases such as HIVE (Erickson et al., 2012). *In vitro* studies employed brain endothelial cell cultures activated with the pro-inflammatory cytokines, tumor necrosis factor-alpha (TNF-α), and interleukin-1beta (IL-1β), caused selective up-regulation of MMP-9 activity, but no significant changes were observed in the MMP-2 or TIMP-2 levels. This proves that MMP-9 is a predominant MMP in neuroinflammatory processes that leads to BBB leakage and disruption.

The inhibition of MMP-9 activity using delivery of short interfering RNA (siRNA) molecules at BMVECs will have a major impact on lowering the BBB permeability. However, a major limitation of this approach is to safely and efficiently deliver siRNA molecules to their target. In addition, free form of siRNA molecules has a very short half-life in physiological conditions that is due to their vulnerability for degradation through endogenous nucleases process. Therefore, they need to be integrated with nanoparticle that will not only protect them from degradation in the biological environment, but also able to direct them to targeted cells or tissues and subsequently facilitate their cellular entry.

In a recent study, we have evaluated the specificity and efficiency of QD complexed with MMP-9-siRNA (nanoplex) in down-regulating the expression of MMP-9 gene in BMVEC that constitute the BBB (Bonoiu et al., 2009). Our results show that silencing of MMP-9 gene expression resulted in the up-regulation of ECM proteins like collagen I, IV, V, and a decrease in endothelial permeability, as reflected by increase of transendothelial resistance (TEER) across the BBB in a well validated *in vitro* BBB model. MMP-9 gene silencing also resulted in an increase in expression of the gene tissue inhibitor of metalloproteinase-1 (TIMP-1). This indicates the importance of a balance between the levels of MMP-9 and its natural inhibitor TIMP-1 in maintaining the basement membrane integrity. These studies promise the application of a novel QD based siRNA delivery system in modulating the MMP-9 activity in BMVECs and other MMP-9 producing cells. This strategy will serve as a platform to design clinically useful QD formulation for preventing neuroinflammation and maintain the integrity of the BBB.

### TRANSCYTOSIS OF BIOCONJUGATED QUANTUM DOTS THROUGH THE *IN VITRO* MODEL OF BBB

**FIGURE 4:**
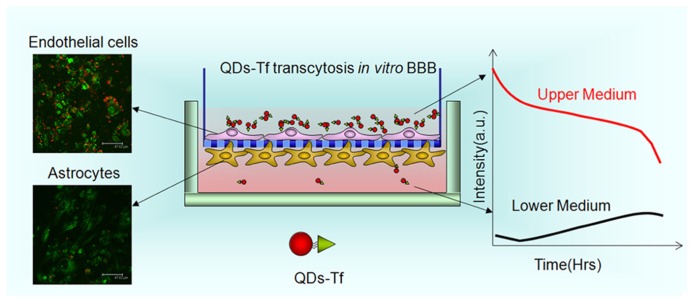
**Visulizing the trancytosisi of QDs-Tf across BBB real-time.** The endothelial cells and astrocytes were imaged by laser scanning confocal microscope to confirm the uptake of QDs by both cells. The fluorescent intensity of the culture media were collected and detected to evaluate the transmigration of QDs.

Recently, we have demonstrated the use of QD bioconjugates as efficient targeted probes for transmigration across the BBB (Bharali et al., 2005; Lucey et al., 2005; Qian et al., 2007). A validated endothelial and astrocytic co-culture BBB model was used to determine the transmigration of QD bioconjugates across the BBB. Since the transferrin receptor protein is highly localized on the endothelial surface of the brain, transferrin was selected to trigger receptor-mediated transport across the BBB. Tf-conjugated QD formulation was prepared and employed for migrating across the *in vitro* BBB model via receptor-mediated transport mechanism (Xu et al., 2008). It was discovered that the migration rate of Tf-conjugated QDs crossing the *in vitro* BBB is both concentration- and time-dependent (**Figure 4**). In our study, following overnight incubation of the Tf-QD bioconjugates with media in the upper chamber of the BBB, the BBB permeability was assayed by quantifying the fluorescence intensity of the media in the upper and the lower chamber of the model, using confocal imaging of both the upper and lower sides of the PET membrane. From the QD fluorescence observed from the lower media, one can see that a portion of the QD-Tf bioconjugates has traversed across the BBB, which is not the case for the control, unconjugated QDs. In addition, confocal microscopic analysis has revealed QD staining on both the upper and lower sides of the PET membrane following treatment with Tf-QD bioconjugates, providing strong evidence for the successful traversing of the functionalized QDs. In the case of treatment with the unconjugated QDs, on the other hand, the QD staining is only observed in the upper side (mimicking the “blood”-end), and not in the lower side (mimicking the “brain”-end), of the PET membrane. These studies provide support that QDs can be directed to cross the blood–brain barrier using our *in vitro* model.

In addition, tight-junction protein Claudin-4 was used concurrently to validate the multiplex imaging technique and also to evaluate quasi-quantitatively the comparative transmigration efficiency and specificity of Tf and anti-Claudin 4 antibody (aC4), by conjugating them with red and orange emitting QDs, respectively. The results showed higher transmigration efficiency of Tf-conjugated QDs over the aC4-conjugated ones across the BBB. This work shows for the first time that the QD multiplexing technique is employed to compare the targeting efficiency of different specific molecules across the BBB. These results illustrate a QD-based platform that will not only allow a direct visualization and quantification of the transmigration ability of various kinds of biomolecules across the BBB, but also facilitate the development of novel diagnostic and therapeutic nanoprobes for early diagnosis and therapy of various disorders of the brain following systemic administration.

### QDs ENHANCING THE DELIVERY OF ANTIRETROVIRAL DRUGS ACROSS THE *IN VITRO* MODEL OF HIVE

**FIGURE 5:**
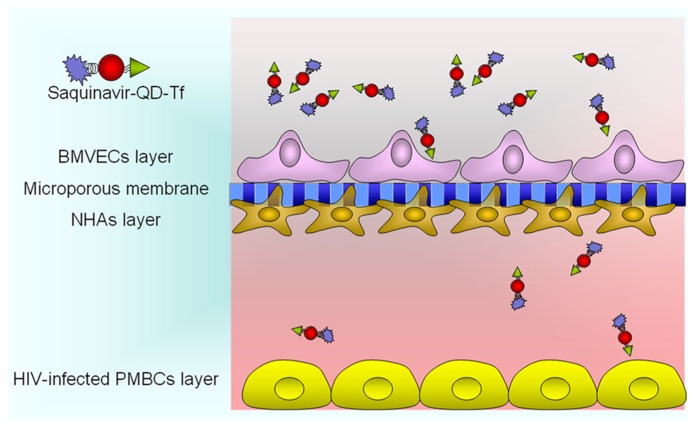
**The therapy of QDs-based nanoplex for *in vitro* HIV-associated encephalopathy model**.

Highly active antiretroviral therapy (HAART) drugs applied for HIV-infected therapy has significantly improved the prognosis of patients. However, the antiretroviral drugs cannot eradicate the HIV virus in the brain because of their poor permeability across the BBB. Consequently, HIV virus in the brain continue to replicate and the HIVE process keep on augmenting, even when the level of HIV-1 virus is too low to be detectable in the peripheral blood. Generally, the HIVE was treated by exposing the whole body to multiple drugs at high doses, resulting in enormous side effects, including the multi-drug resistance. Thus, development of targeted approaches or increasing the drug-delivery efficiency will improve the safety and efficacy of HAART by reducing the dosage and adverse effects. Thus, the theranostic QDs have been prepared by conjugating the fluorescent QDs with the targeting molecule transferrin and the HAART drug Saquinavir, which is the first protease inhibitor approved by the Food and Drug Administration (FAD) of United States (Mahajan et al., 2010). In order to shorten the experimental cycle and reduce the cost, a HIVE *in vitro* model has been established. Besides the BBB *in vitro* model as in Section “Transcytosis of Bioconjugated QDs Through the *IN VITRO* Model Of BBB,” HIV-1 infected peripheral blood mononuclear cells (PBMCs) are cultured on the bottom of the lower chamber (basolateral end), as **Figure 5**. The concentration of Saquinavir used in this work was 10 and 40 nM, within the effective concentration range of this drug.

The results demonstrated the nanoplex did not change the integrity of the BBB in HIVE *in vitro* model, as assessed by the TEER value during transversing across the BBB. Significant decrease in p24 production and LTR/RU5 gene expression in the HIV-1 infected PBMCs was observed, which verified that the antiviral efficacy of Saquinavir was enhanced when delivered by the targeted nanocarrier across the BBB. Our results demonstrated that the QDs-Tf-Saquinavir nanoformulation increases the drug solubility, enhance systemic bioavailability, and the excellent optical properties of QDs also visualizes the distribution and accumulation of nanoplexes in brain. In another previous work, we used the same strategy to construct a novel theranostic nanoformulation by conjugating HAART drug Amprenavir, QDs, and Tf. The p24 production and LTR/RU5 gene expression in the HIV-1 infected PBMCs were decreased significantly as well (Mahajan et al., 2012).

Both the studies mentioned above demonstrated that the QDs-Tf-HAART drugs nanoformulation can transverse the BBB and significantly inhibit HIV-1 replication in the infected PBMCs. Such primary results offered the basis to develop novel QD based nanoplex with targeting molecules and HAART drugs, which could enhance the drug delivery efficacy across the BBB and facilitate the uptake of the nanoplex by HIV-1 infected cells in the brain.

The application of nanotechnology provides unprecedented opportunities for addressing many of the gaps in the diagnosis and therapy of diseases. Nanoparticle technology offers a significant advancement in the ability to increase drug translocation across the BBB. The development of BBB permeable, multifunctional drug-loaded nanoparticles will provide a significant advancement toward the therapy of neurological disorders associated with HIV-1; further, these nanoparticle systems will integrate high-resolution imaging capability in addition to therapeutic modalities. We have demonstrated the ability of a Tf-QD-Amprenavir nanoplex to transverse the BBB and significantly inhibit HIV-1 replication in HIV-1-infected monocytes, demonstrating their anti-HIV-1 efficacy in the brain. The use of such nanotechnology platforms for delivery of antiretroviral drugs will revolutionize the treatment of neuro-AIDS.

## SUMMARY AND FUTURE OUTLOOK

This review discusses the potential use of QD bioconjugates for sneaking of drugs and/or imaging agents across the BBB, that would allow simultaneous imaging and therapy in targeted areas of the brain. The successfully engineered transmigrational QDs *in vivo* would allow groundbreaking technological advances in functional imaging and therapeutic approaches for the diagnosis and treatment of several brain diseases such as brain tumor, stroke, neuro-AIDS, and neurodegenerative disorders. The BBB is one of the major physiological “conundrums,” which on one hand prevents the brain from damage by unwanted blood-borne pathogens and toxins, whereas on the other hand impedes the systemic delivery of diagnostic and therapeutic agents in the CNS. In addition to significantly advancing the diagnosis and therapeutic technologies within the CNS, this review also highlights the basic scientific mechanism of transport across the BBB, which would provide useful guidelines for other researchers to bolster the drug discovery program aimed at CNS therapeutics. This QD technology for migrating across BBB could potentially transform into personalized medicine. The translational impact of this technology lies in the drug delivery across the BBB and further engineered QD formulations integrated with therapeutic drugs and molecules for tissue specific recognition and sustained release of the therapeutic drug from the nanoparticles once it reaches its target tissue. In the long term, this developed technology will be generalized toward the development of advanced diagnostic/therapeutic strategies for treating neurological disorders.

Despite the encouraging result on using QDs for drug delivery across the BBB, there remains a serious concern about the toxicity of the QDs *in vivo* that is mainly originated from the intrinsic potential toxic nature of the semiconductor materials themselves (Won et al., 2009). To date, different strategies have been created to reduce the toxicity and side effects of QDs, including coating the QDs with biocompatible polymer and synthesizing the cadmium-free QDs that is based on silicon or carbon materials (Hardman, 2006; Erogbogbo et al., 2008). We believe that these proposed strategies will allow one to fabricate QDs with minimum toxicity and employ them for neurological pharmaceutical applications.

## Conflict of Interest Statement

The authors declare that the research was conducted in the absence of any commercial or financial relationships that could be construed as a potential conflict of interest.
